# PEP-EDIT: a web server for the 3D generation and interactive editing of complex peptides

**DOI:** 10.1093/nar/gkag455

**Published:** 2026-05-14

**Authors:** Nicolas Chevrollier, Alexis Dougha, Celine Ye, Dirk Stratmann, Gautier Moroy, Julien Rey, Samuel Murail, Pierre Tufféry

**Affiliations:** Université Paris Cité, CNRS, Inserm, Unité de Biologie Fonctionnelle et Adaptative, RPBS, F-75013 Paris, France; Université Paris Cité, CNRS, Inserm, Unité de Biologie Fonctionnelle et Adaptative, RPBS, F-75013 Paris, France; Université Paris Cité, CNRS, Inserm, Unité de Biologie Fonctionnelle et Adaptative, RPBS, F-75013 Paris, France; Université Paris Cité, CNRS, Inserm, Unité de Biologie Fonctionnelle et Adaptative, RPBS, F-75013 Paris, France; Sorbonne Université, UFR 925, F-75006 Paris, France; Université Paris Cité, CNRS, Inserm, Unité de Biologie Fonctionnelle et Adaptative, RPBS, F-75013 Paris, France; Université Paris Cité, CNRS, Inserm, Unité de Biologie Fonctionnelle et Adaptative, RPBS, F-75013 Paris, France; Université Paris Cité, CNRS, Inserm, Unité de Biologie Fonctionnelle et Adaptative, RPBS, F-75013 Paris, France; Université Paris Cité, CNRS, Inserm, Unité de Biologie Fonctionnelle et Adaptative, RPBS, F-75013 Paris, France

## Abstract

In recent years, the development of peptide drugs has seen significant growth. These molecules often go beyond simple linear chains composed of the standard 20 amino acids. Peptide drugs frequently incorporate non-standard amino acids, non-amino components, and can exhibit mono- or multicyclic structures, branching, and other complex topologies. Consequently, there is a growing need for accessible tools that allow researchers to easily generate and modify 1D, 2D, and 3D representations of these complex peptides, serving as a starting point for further optimization. PEP-EDIT was created to meet this need. It offers a user-friendly, interactive web interface for generating complex peptide representations from 1D BILN (Boehringer Ingelheim Line Notation) sequences, using a customizable monomer library. Building on the pyPept library, PEP-EDIT enhances its functionality with options such as pH-dependent protonation and simplified specification of conformational constraints. The platform leverages interactive 2D and 3D visualizations to guide peptide design, offers intuitive management of monomers and 3D models, and includes collaborative and interactive visualization tools. PEP-EDIT is available at https://pep-edit.rpbs.univ-paris-diderot.fr. This website is free and open to all users and there is no login requirement.

## Graphical Abstract

**Graphical Abstract:**
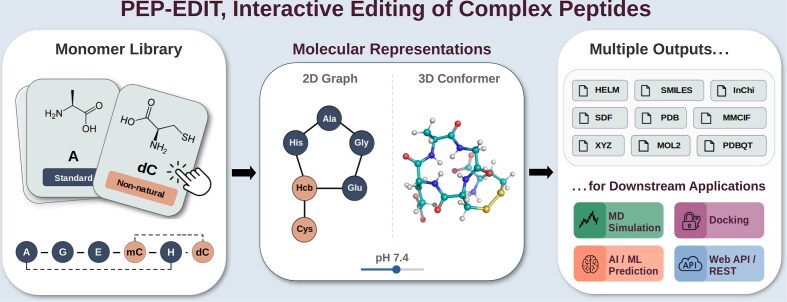

## Introduction

In the recent years, peptides have emerged as powerful drug candidates, with blockbusters like Ozempic and Mounjaro estimated to generate over $50 billion in sales in 2025 [1]. Peptide drugs rarely rely solely on the 20 standard amino acids. For instance, Ozempic and Mounjaro incorporate a fatty acid chain and non-standard amino acids like the aminoisobutyric acid. Other peptides feature cyclic (e.g. cyclosporin and pasireotide) [2, 3] or branched structures [4], making their structures and topologies sometimes complex.

Creating and editing 3D representations of complex peptides for *in silico* drug development remains a significant challenge. Peptides often contain far more atoms than small molecules, making traditional atom-based descriptions developed for small compounds cumbersome to handle. At the same time, complex peptides can defy the simple linear assembly seen in standard proteins, difficulties arising from the occurrence of non-standard amino acids, exotic cycles, branching, and other intricate topologies. Fortunately, recent advances in peptide notation languages such as HELM [5, 6] and the more streamlined BILN (Boehringer Ingelheim Line Notation) [7] have addressed this bottleneck. BILN, in particular, simplifies the description of peptides composed of standard, non-standard, or exotic monomers, adopting linear, cyclic, or branched topologies in the form of a 1D representation that is fairly understandable by humans. Complex peptides made of several peptide chains linked together by other bonds than the peptide bond are also handled.

Yet, having a way to describe complex peptides is just the first step. Generating realistic 3D conformations also requires creating an initial 3D model that meets all the constraints of their intricate topologies, providing a foundation for further refinement and a resource for design. Clearly, there is a need for easy-to-use tools to generate 3D models of complex peptides.

Whereas numerous tools to predict peptide properties have been setup [8], fewer tackle the issue of the initial 3D generation. Some desktop applications like Rosetta can since long handle non-standard amino acids [9]. Some others like SAMSON (https://www.samson-connect.net/) and PICKAPEP [10] can edit complex molecules, including modified peptides. However, freely accessible online tools for 3D complex peptide generation remain scarce. Ketcher (https://lifescience.opensource.epam.com/ketcher/), an online tool internally using the HELM notation, can generate 3D conformers for small compounds and employs a monomer library for larger ones, but currently only produces 2D representations for linear and cyclic peptides. PEP-DRAW’s free online server (https://pepdraw.com/) offers only basic functionality. Among protein/peptide-oriented software, AlphaFold3’s command-line mode supports complex peptide generation, but its web server is limited to standard amino acids and selected post-translational modifications. The Boltz [11] web server can also handle the non-standard amino acids of the Chemical Component Dictionary (CCD) [12], but it is available only to registered users, similarly to the Chai-1 web server [13]. Among specialized peptide structure prediction freely available web servers like PEP-FOLD [14] or pepstrmod [15], only the latter accepts d- and non-standard amino acids, yet it cannot handle more exotic residues or generate complex cyclic or lipidated peptides.

PEP-EDIT is an online platform designed to generate 3D coordinates for complex peptides, to serve as a starting point for further studies, including conformation refinement, sampling, and analyses. It elaborates over the pyPept library [16] that offers tools for generating starting 3D conformations of complex peptides starting from FASTA, HELM, and BILN representations. pyPept leverages a monomer library in SDF format [17], integrates with RDKit for cheminformatics (Landrum G, RDKit: Open-source cheminformatics; https://www.rdkit.org), and includes a secondary structure predictor. It also features converters between HELM, BILN, and FASTA, enabling seamless 3D model generation using RDKit. While pyPept is freely available, it requires local installation and command-line operation, and its use can reveal difficult to the non-expert user. PEP-EDIT offers a user-friendly web interface for defining BILN sequences from a user extendable monomer library, complemented by interactive 2D and 3D visualizations to guide peptide design. The platform also extends pyPept’s functionality with several key features: database-oriented monomer library management, pH-dependent protonation, simplified conformational constraint specification, intuitive monomer and 3D template management, and collaborative, interactive visualization tools.

## Materials and methods

### The PEP-EDIT pipeline

**Figure 1.**
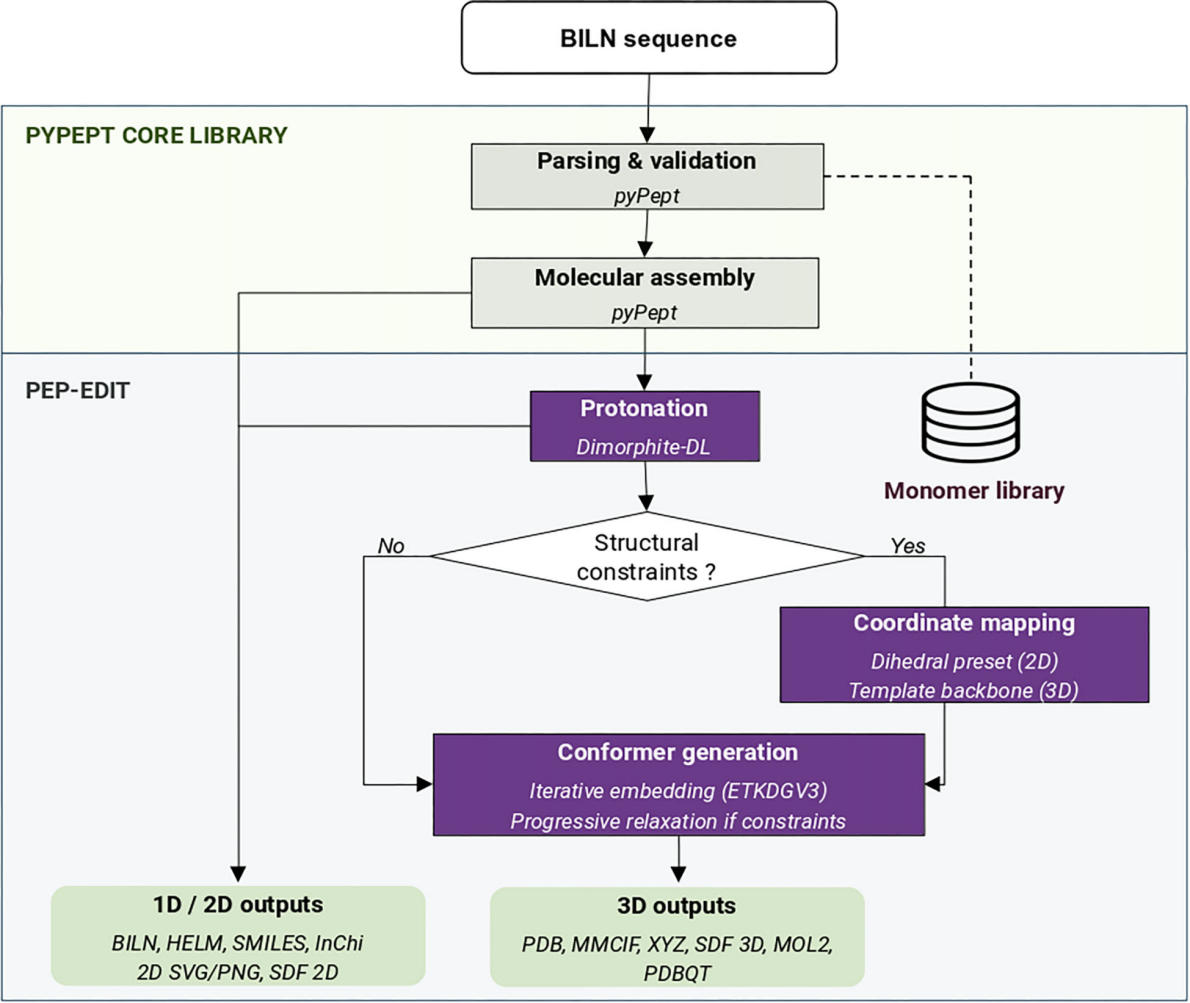
PEP-EDIT processing pipeline. A BILN sequence is parsed and assembled into a molecular graph using the pyPept core library (gray), with monomer definitions resolved from a MongoDB-backed library. PEP-EDIT extensions (purple) add pH-dependent protonation via Dimorphite-DL, optional coordinate mapping from secondary-structure presets or PDB template backbones, and iterative ETKDGv3 conformer generation with progressive constraint relaxation. The pipeline produces both 1D/2D representations and 3D structural outputs.

A brief overview of PEP-EDIT processing pipeline is reported in Fig. 1 and is detailed in the following sections.

### Sequence input, parsing, and molecular assembly

PEP-EDIT uses the BILN format [7] as its internal representation. Users may input sequences directly in BILN or import FASTA or HELM [5] notation. The input sequence is parsed and validated using a modified version of pyPept [16], which resolves each monomer symbol against a MongoDB-backed monomer library. The validated sequence is then assembled into a complete RDKit (https://www.rdkit.org) molecular graph by joining monomer substructures according to their R-group attachment definitions. PEP-EDIT provides a public monomer library of 324 entries originally distributed with pyPept, with minor corrections to monomer classifications and protonation-state compatibility.

### Protonation

Following molecular assembly, pH-dependent protonation is performed using a modified version of Dimorphite-DL [18] at a user-specified pH (default 7.4). Selected SMARTS pKa definitions have been adjusted to better match known amino acid behavior at physiological pH (e.g. neutral phenol, imide, and amide groups). The protonated molecular graph serves as the common starting point for both two-dimensional depiction and three-dimensional conformer generation.

### Two-dimensional depiction and 1D/2D outputs

Two-dimensional coordinates are computed using RDKit’s depiction algorithms. The SVG output is semantically annotated at the residue and bond levels, enabling interactive highlighting and topology-aware manipulation in the browser. Alongside the 2D depiction (SVG and PNG), PEP-EDIT returns the BILN and HELM notations, the canonical isomeric SMILES, InChI, and a 2D SDF file with normalized coordinates.

### Three-dimensional conformer generation

PEP-EDIT generates single three-dimensional conformers using RDKit’s ETKDGv3 distance geometry method [19, 20]. Three generation modes are supported.

In *de novo* mode, no spatial constraints are applied; the conformer geometry is determined by the molecular topology and ETKDGv3’s built-in knowledge terms.

When structural constraints are specified, a coordinate-mapping step is performed prior to embedding. In secondary-structure-guided mode, users specify per-residue secondary structure using DSSP-format codes (H/E/–). Backbone φ/ψ dihedral angles are preset to canonical Ramachandran values (−57°/−47° for α-helices, −120°/120° for β-strands), with angle mirroring for d-amino acids. In template-guided mode, backbone atom positions are extracted from an experimentally determined PDB structure (provided by PDB identifier or direct upload). User-defined chain selection, residue ranges, offsets, and per-residue masking allow flexible mapping between the template and the target peptide, enabling constrained generation where conserved regions adopt the template fold while modified positions are resolved *de novo*. In both cases, the mapped coordinates serve as spatial constraints during embedding.

For all modes, PEP-EDIT employs an iterative embedding strategy with progressive relaxation, inspired by constrained conformer generation approaches used in macromolecular modeling [21]: embedding is attempted first with 100% of constraints enforced, then with randomly sampled subsets (90%, 80%, 50%) using increasing numbers of attempts at each stage. This empirically determined schedule addresses the geometric conflicts that arise when external coordinate constraints cannot be perfectly reconciled with the molecule’s inherent bonding requirements. The same multi-attempt strategy is applied without constraints in *de novo* mode to maximize embedding success. The first successful embedding terminates the procedure.

### 3D outputs

The conformer generation step produces structural outputs in multiple standard formats: PDB (with residue and chain annotations), SDF with 3D coordinates, XYZ coordinate block, and MDL Molblock. The pipeline additionally exports Tripos MOL2, AutoDock PDBQT (with Gasteiger partial charges), and mmCIF formats via Open Babel [22]. A PNG snapshot of the 3D structure from the Mol* Viewer [23] is also provided. All 3D conformers are displayed interactively in Mol* with support for multiple representations, residue labeling, and overlay of PDB scaffold templates for visual comparison. All output files can be downloaded individually or as a single ZIP archive.

### Architecture implementation

PEP-EDIT is a client–server web application. The server-side backend is built around a Python Flask REST API. Persistent data—including monomer definitions, session metadata, and conformer job records—are stored in MongoDB. Three-dimensional conformer generation is delegated to Celery workers using Redis as message broker, enabling asynchronous execution with real-time progress reporting. The client-side frontend is a React single-page application styled with Material UI and Tailwind CSS. The source code is available at https://gitlab.rpbs.univ-paris-diderot.fr/rpbs/pypept-api.

### The PEP-EDIT web server interface

**Figure 2.**
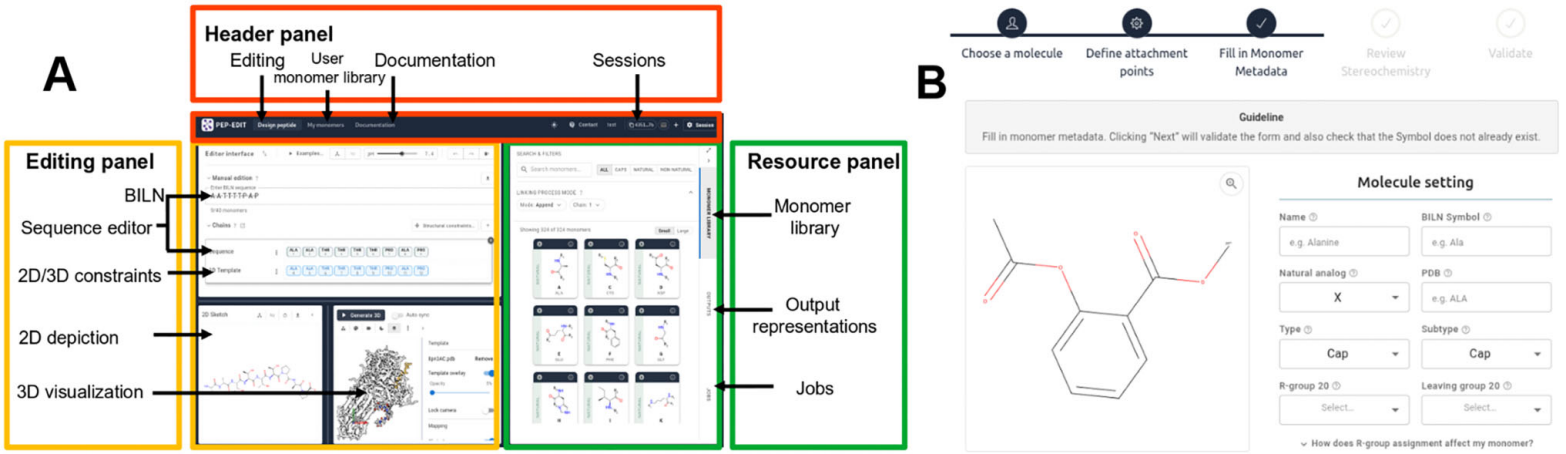
(**A**) Organization of the PEP-EDIT web server interface. (**B**) Organization of the monomer editing facility.

The PEP-EDIT web server interface is organized as three main panels, as illustrated in Fig. 2 . Peptide sequences are entered and edited in BILN notation through a live text field. The sequence editor supports multichain editing, drag-and-drop residue reordering, bond linking and breaking modes, and undo/redo (up to 20 steps). Structural constraints can be specified per residue as secondary structure states (helix, strand, coil) or imported from a PDB/mmCIF scaffold template with residue-level mapping control. Protonation states are computed at a user-defined pH (default 7.4). Fifteen preset examples spanning linear, cyclic, capped, and constrained peptides are provided to help new users get started.

The three molecular views, sequence editor, 2D depiction, and 3D viewer, are fully synchronized: edits to the BILN sequence trigger live updates of both the 2D sketch and the available 1D/2D output formats, while monomer hover events are propagated across all three panels in real time. The 2D depiction supports interactive bond creation and removal through a graphical link/unlink mode. The 3D viewer, powered by Mol*, offers multiple representations (cartoon, ball-and-stick, surface, licorice, among others), adjustable color schemes, residue labels, and overlay of a PDB scaffold template for visual comparison. For short peptides (<8 monomers), 3D conformer generation is triggered automatically; for larger constructs, it is launched on demand to preserve responsiveness.

The resource panel provides three tabbed views: a searchable monomer library (filterable by category: standard amino acids, non-natural residues, and capping groups), an output panel offering 12 export formats, and a job history listing all conformer generation runs with their status, from which any previous result can be fully restored.

Each monomer in PEP-EDIT is defined at the chemical–structure level as a molblock (the core data structure of the SDF format), annotated with attachment points and metadata, and referenced in BILN sequences by its symbol. BILN thus operates as a sequence-level language to describe peptide topology and connectivity, while SDF provides the underlying monomer chemistry. Beyond the built-in library of 324 monomers, users can register personal monomers through two routes: (i) a guided wizard, in which the user enters a SMILES string, interactively selects bonds to define attachment points, assigns a BILN-compatible symbol and metadata, and obtains a validated monomer definition ready for immediate use in sequences; or (ii) direct import from a PEP-EDIT-compatible SDF file, in which each record must include properly defined attachment points (leaving groups/R-groups), a monomer symbol, and the required metadata attributes. Multi-record SDF files are supported, and personal monomer collections can be exported and reimported as SDF. Users may also submit monomers for inclusion in the public library via a moderated review process.

PEP-EDIT uses anonymous, ID-based sessions that persist across visits and require no account creation. Sessions encapsulate personal monomers and job history, and can be shared by sending the session identifier to collaborators. Additionally, a dedicated instance of PEP-EDIT is deployed within an n.eko environment (https://neko.rpbs.univ-paris-diderot.fr/?usr=guest&pwd=rpbs), enabling synchronous collaborative editing in a single shared session where all user actions are propagated in real time across connected clients. This setup supports remote collaboration and interactive teaching scenarios.

## Case studies

The following paragraphs briefly illustrate the use of PEP-EDIT for varied tasks.

### Generating representations of the epidermin

Epidermin belongs to the class of polycyclic peptide antibiotics called lantibiotics, which is the abbreviation for lanthionine-containing peptide antibiotics [24]. Its antibiotic activity is due to the pore formation in the bacterial cell membranes. However, the exact mechanism is complex and has not been fully elucidated [25]. Epidermin is synthesized by ribosomes and undergoes post-translational modifications during the maturation to produce its biologically active form. It is composed of 21 amino acids, including 2 non-proteinogenic amino acids, the 2-aminoisobutyric acid and the dehydroalanine, respectively. Moreover, epidermin has four sulphide rings, consisting of two meso-lanthionine, one (2S, 3S, 6R)-3-methyllanthionine, and the unusual ring structure containing S-[(Z)-2-aminovinyl]-d-cysteine.

**Figure 3.**
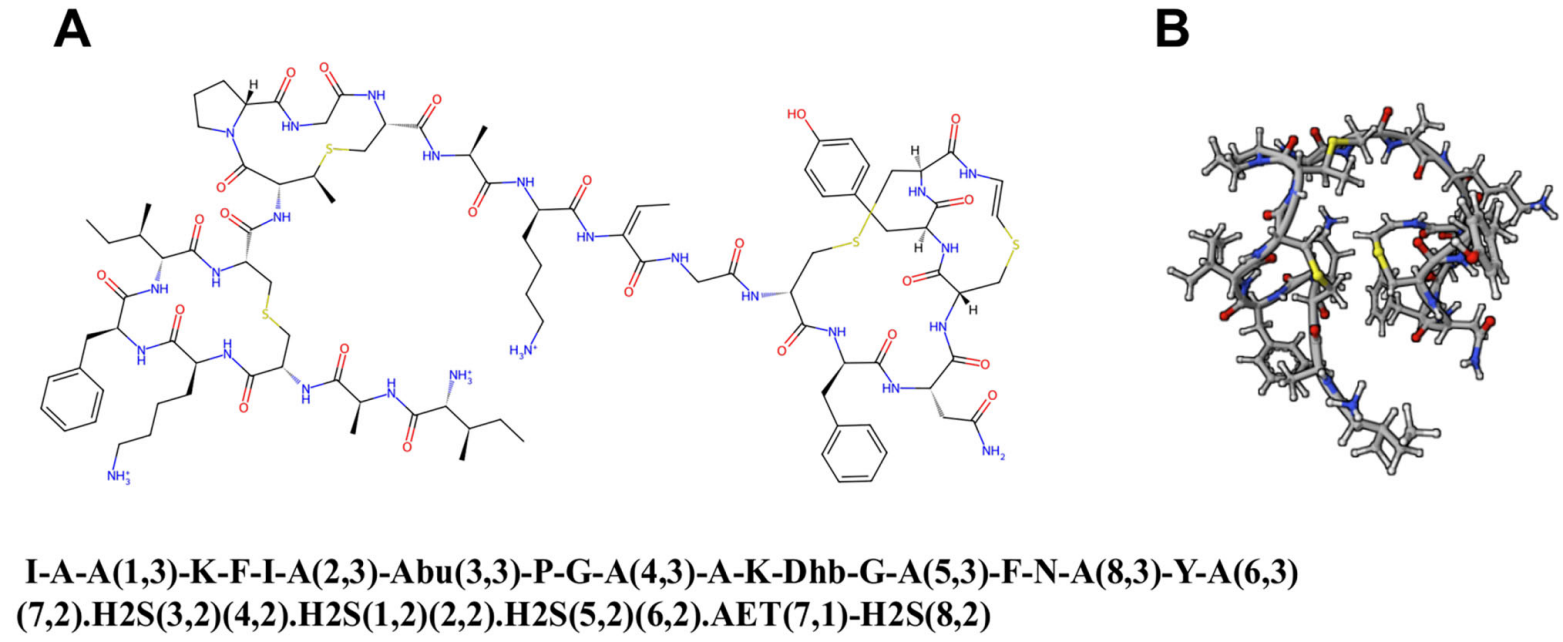
2D (**A**) and 3D (**B**) representations of epidermin as generated by the PEP-EDIT web service. The BILN sequence is reported.

To build the epidermin (Fig. 3), we first had to define some missing monomers using the PEP-EDIT “My monomers” functionality: a modified alanine (Ala) and a modified 2-aminobutanoic acid (Abu) both capable of forming a bond with the methyl group of their side chain, a hydrosulfuric acid (H_2_S), and a 2-aminoethenol (AET) monomers for the bridges. These monomers have since been propagated to the PEP-EDIT public monomer library. The modified alanine at positions 3 and 7 have then been linked through an H_2_S monomer, and the 2-aminobutanoic acid and alanine at positions 8 and 11, respectively, have been linked with an H_2_S monomer. Finally, a 2-aminoethenol (AET) monomer has been used to form the last ring between the alanine corresponding to Ala 19 and the carboxyl-terminus group of Ala 21.

### Editing a peptide in interaction with a partner protein

The BCL-2 family proteins regulate the intrinsic cell death pathway through protein–protein interactions to control mitochondrial outer membrane permeabilization [26]. Among them, several members act as anti-apoptotic proteins, while others trigger apoptosis. The interactions between BCL-2 family proteins are therefore intensively studied in order to design therapeutic molecules able to restore apoptosis in cancer cells.

BCL-x_L_, an apoptotic protein, is able to interact with BAD, a pro-apoptotic protein, through its BH3 domain. The structures of the complex between BCL-x_L_ and the BAD fragment of the BH3 domain is known (PDB ID: IG5J) [27]. The BAD peptide must adopt an α-helix structure to bind to BCL-x_L_. Several studies have been performed to constraint the peptide in α-helix structure.

**Figure 4.**
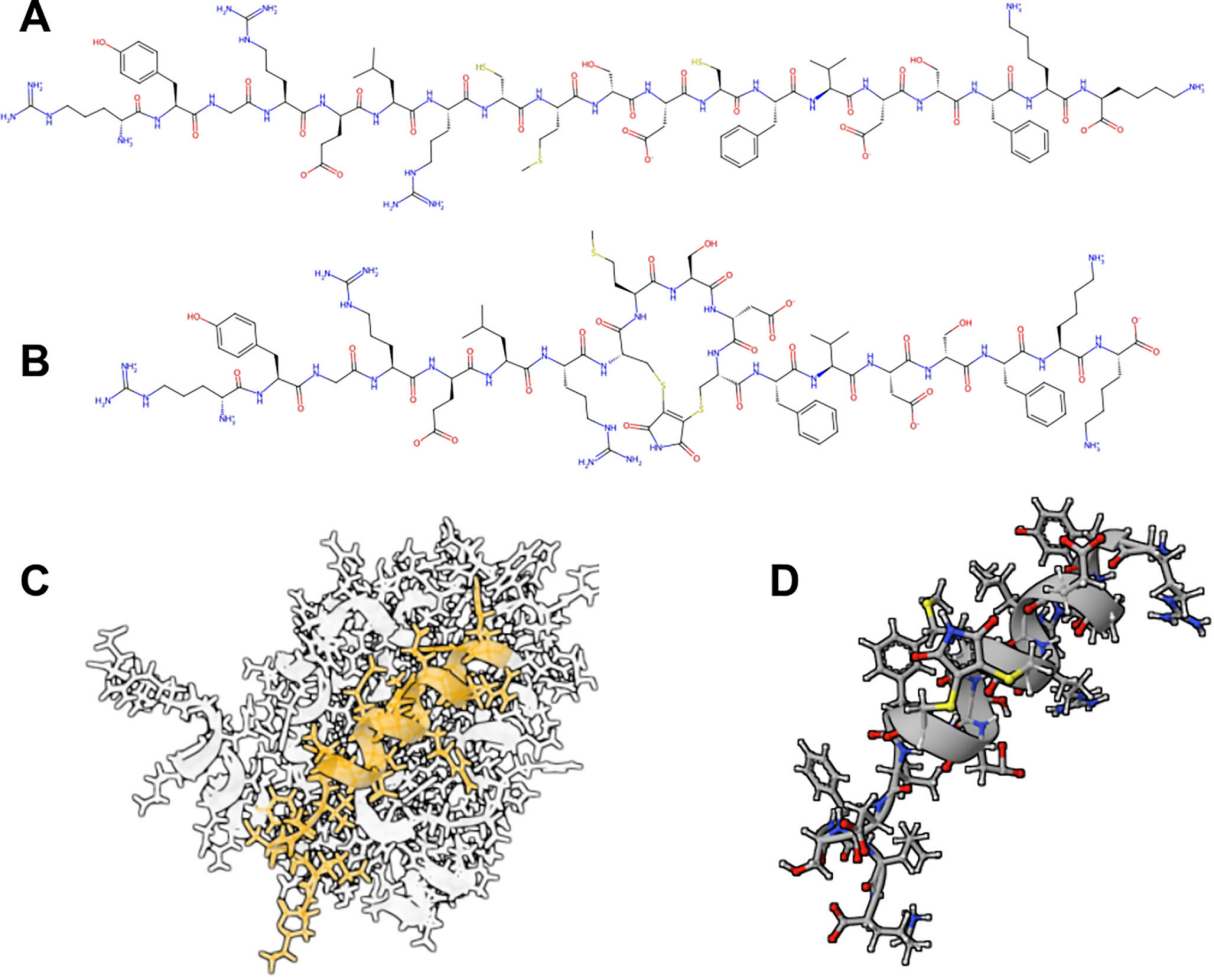
2D representation of the RYGRELRCMSDCFVDSFKK peptide, linear (**A**) and constraint (**B**) with the maleimide group between two cysteine residues. (**C**) 3D representation of the peptide, in yellow, that is the template for the RYGRELRCMSDCFVDSFKK peptide and, in white, BCL-x_L_ protein. (**D**) The peptide with the maleimide group between two cysteine residues and constrained to be in an α-helix.

Using PEP-EDIT, constraints such as the maleimide group can easily be imposed to stabilize the α-helix structure [28]. The RYGRELRCMSDCFVDSFKK peptide (Fig. 4A), one of the peptides studied by Zhang and colleagues [28], has been used as an example to demonstrate the “structural constraints” functionality of PEP-EDIT. The α-helical structure has been imposed using chain B of the IG5J PDB structure as a template with a six-amino-acid shift sequence to maintain sequence homology between the studied peptide and the peptide in the PDB complex (Fig. 4C). The maleimide group was created and linked to the two cysteine residues in the peptide (Figs 4B). The resulting peptide was generated in 3D, taking into account the constraint of being in an α-helix (Fig. 4D).

### PEP-EDIT generated conformation as input for molecular dynamics or quantum mechanics simulations

**Figure 5.**
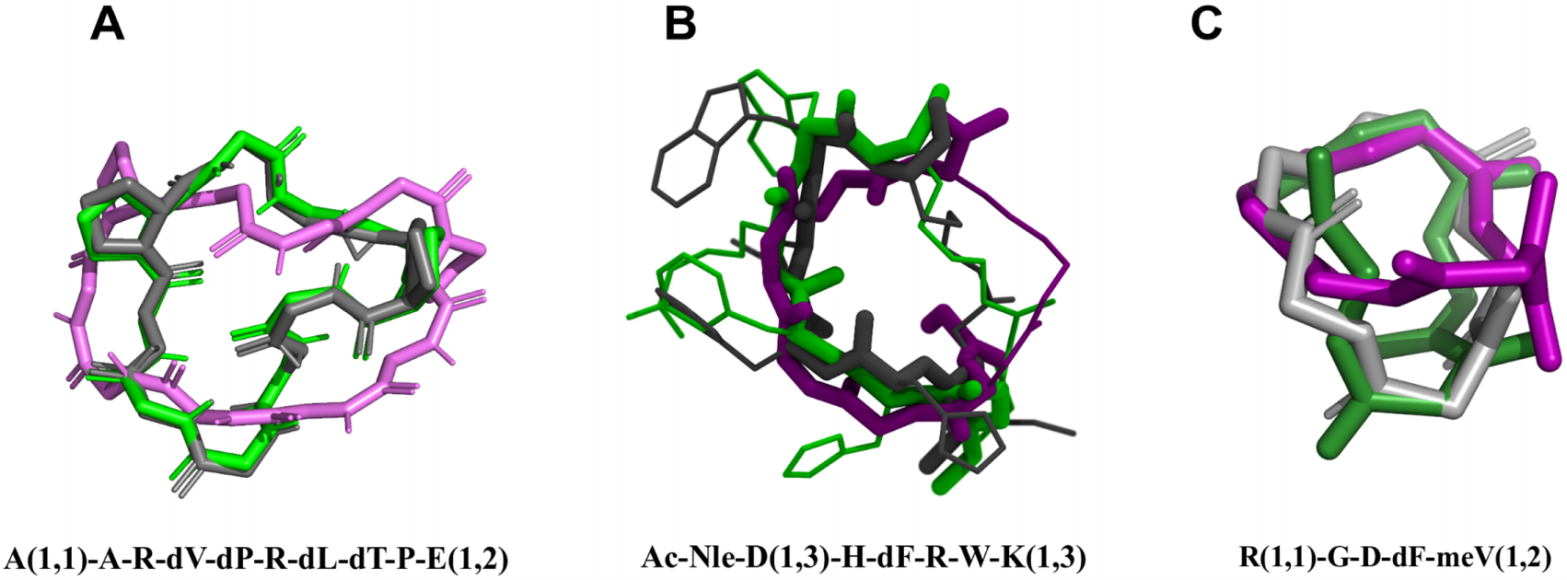
**A**) REMD refinement of the PEP-EDIT structure for one head-to-tail cyclic peptide of 10 amino acids recently studied using coarse grained simulation [31]. The 3D starting structure obtained from PEP-EDIT has a backbone RMSD of 3.2Å to the reference structure. The representative structure of the highest populated cluster has a backbone RMSD of only 0.3 Å. (**B**) ST refinement using the OpenFF force field of the PEP-EDIT structure of bremelanotide. The backbone atoms of the initial 3D model generated by PEP-EDIT (2.2 Å backbone RMSD to the reference structure) are shown as purple sticks. The ST-refined conformation exhibiting the minimal backbone RMSD (1.2 Å) to the experimental structure is shown in green sticks. The reference bound structure (PDB ID 7F55 [30]) is displayed in gray sticks. (**C**) Best conformer generated by ORCA’s GOAT algorithm for the orphan cyclic peptide drug cilengitide [33, 34]. In this example, we used the semi-empirical method GFN2-xTB [35] with the ALPB solvation model [36] but more expensive methods are expected to yield even more accurate results. The initial deviation of the PEP-EDIT-generated conformation to the experimental bound reference (PDB 1L5G) is 1.41Å while ORCA enabled to sample more conformations with the best one achieving 0.82Å RMSD. Structure color code: gray: experimental structure; purple: 3D starting structure as generated by PEP-EDIT; green: best structure after conformational sampling. The BILN sequences of the peptides are reported below the structures.

To demonstrate the versatility of PEP-EDIT-generated conformations for modeling, we evaluated their use in enhanced molecular simulations and quantum mechanical refinement. For the cyclic peptide bremelanotide [a melanocortin receptor agonist approved for the treatment of hypoactive sexual desire disorder [29], BILN: Ac-Nle-D(1,3)-H-dF-R-W-K(1,3)], simulated tempering (ST) (10 µs, OpenFF force field) enabled extensive exploration of the conformational landscape, reaching a minimal backbone RMSD of 1.2 Å relative to the experimental bound structure (PDB ID 7F55) [30] (Fig. 5B) and identifying near-native states among the dominant solution clusters despite simulating the unbound peptide. Major conformational states were sampled within the first ∼2 µs, indicating rapid convergence. In addition, short REMD simulations (50 ns) on a cyclic peptide of 10 amino-acids [BILN: A(1,1)-A-R-dV-dP-R-dL-dT-P-E(1,2)] using a coarse-grained protocol recently published [31] were sufficient to recover sub-angstrom conformations close to experimental references (Fig. 5A), highlighting the efficiency of the workflow. Finally, PEP-EDIT structures can also directly serve as input for QM refinement (e.g. ORCA [32]), where conformational optimization further improves agreement with experimental structures. Figure 5C presents the results obtained for cilengitide [33, 34], a cyclic peptide of five amino acids, where the PEP-EDIT-generated conformation deviates from the experimental one by 1.41Å, whereas QM sampling can further identify conformations at only 0.82Å RMSD. Together, these results demonstrate that PEP-EDIT provides relevant starting models suitable for efficient MD and QM refinement pipelines. Further methodological details are provided in the Supplementary data.

## Conclusion and future work

PEP-EDIT is designed to be a user-friendly and evolving web resource for rapidly generating 1D, 2D, and 3D representations of complex peptides. Its interface is rich and integrates multiple functionalities.

On the one hand, PEP-EDIT offers peptide editing and generation coupled with interactive visualization, featuring synchronized 1D, 2D, and 3D representations. A key strength is its handling of 2D/3D constraints, which facilitates the generation of more realistic 3D conformations compared to those produced by RDKit, which primarily satisfy local geometric constraints. Outputs in various formats also enable the use of generated starting conformations with other tools, as demonstrated in the use cases. On the other hand, by combining public and private monomer libraries, PEP-EDIT extends its utility beyond the current monomer library, providing users with greater flexibility. Finally, PEP-EDIT incorporates collaborative and educational features. Library and session sharing, interface interactivity, and integration with platforms such as n.eko enhance its value for teaching and teamwork.

PEP-EDIT-planned evolutions in the short term aim to address some of its current limitations, such as ensuring the chemical compatibility of non-peptidic bonds generated during cyclization. Connecting the monomer library to the CCD is also desirable, although it raises nomenclature issues. In the medium term, the integration of physicochemical or ADMET property prediction tools could be considered. In the longer term, a more robust interface with 3D conformation refinement tools, including force field parameters for the entire monomer library, may be developed. However, this will require a precise evaluation of the protocols to be used.

## Supplementary Material

gkag455_Supplemental_File

## Data Availability

The PEP-EDIT web server (https://pep-edit.rpbs.univ-paris-diderot.fr) is free and open to all users and there is no login requirement.
