# Supplementary material for: PEP-EDIT: a web server for the 3D generation and interactive editing of complex peptides
Source: Nucleic Acids Res. 2026 May 14;54(W1):W287–94. doi: 10.1093/nar/gkag455 (PMC13355081; doi:10.1093/nar/gkag455)
Supplement: gkag455_Supplemental_File [file gkag455_supplemental_file.pdf]

Supporting Information for

PEP-EDIT: a web server for the 3D generation and interactive editing of complex peptides.

AUTHORS

Chevrollier Nicolas[1], Dougha Alexis[1], Ye Celine[1], Stratmann Dirk[1,2], Moroy Gautier[1], Rey Julien[1], Murail Samuel[1] and Tufféry Pierre[1,*]

[1] Université Paris Cité, CNRS, Inserm, Unité de Biologie Fonctionnelle et Adaptative, F-75013 Paris, France
[2] Sorbonne Université, UFR 925, 4 place Jussieu, 75252 Paris Cedex

* To whom correspondence should be addressed. Email: pierre.tuffery@u-paris.fr

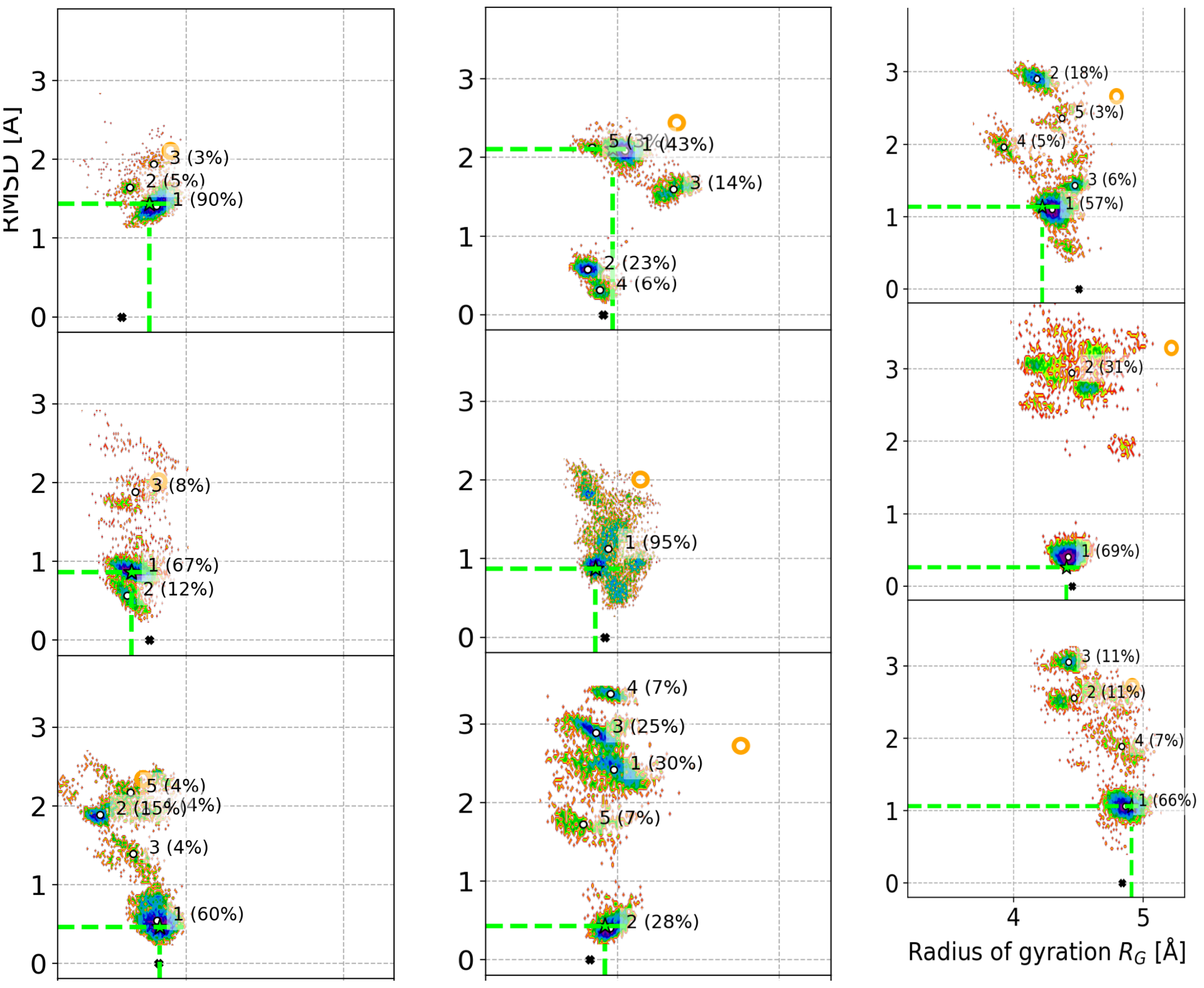

Figure S1: Nine cyclic peptides from (1). First column: peptides of 7 amino acids: 7A, 7B and 7C (nomenclature from (1)). Second column: peptides of 8 amino acids: 8A, 8B and 8C. Third column: peptides of 9 and 10 amino acids: 9A, 10A and 10B. Starting structure from PEP-EDIT in orange circles. Highest populated cluster in green dashes.

PEP-EDIT generated conformations as input for **REMD calculations**

As test set we used the nine cyclic peptides described in our former publication (1). The 3D conformations generated by PEP-EDIT (PDB format) were submitted to a conformational sampling using a Replica-Exchange Molecular Dynamics (REMD) protocol (1). The head-to-tail cyclic peptides have only natural amino acids in L- and D-forms, which allowed us to employ the standard protein force fields Amber14SB. As the original REMD protocol used long trajectories of 1000 ns repeated five times, we took here the opportunity to test if shorter REMD trajectories of only 50 ns can already yield an accurate free energy map and a highest populated cluster near to the experimental reference structure. As can be seen in the **Figure S1** short REMD trajectories can already be sufficient to yield structures below 1Å RMSD to the reference structure, which are in most cases ranked at the first position according to the cluster populations. This demonstrates the possibility to use PEP-EDIT with REMD in a pipeline without much computational extra costs for the REMD part. Here only about 50 CPU hours were required per peptide, without the need of GPU resources as simulations are done in implicit solvent.

- **PEP-EDIT generated conformations as input for MD simulation and OpenFF force field calculation**

Bremelanotide is a melanocortin receptor agonist approved for the treatment of Hypoactive Sexu-al Desire Disorder (2). It is a cyclic peptide of BILN sequence Ac-Nle-D(1,3)-H-dF-R-W-K(1,3). The PDB structure and corresponding SMILES representation generated using PEP-EDIT were used as input for molecular dynamics simulations (scripts are available at github.com/samuelmurail/Pep-Edit_ST). The SMILES string was processed using RDKit (RDKit: Open-source cheminformatics. https://www.rdkit.org) to ensure chemical consistency and proper parameter assignment for non-standard residues.

All simulations were performed using OpenMM 8 (3). The peptide was parame-terized using Open Force Field (OpenFF) toolkit (4). The system was solvated in explicit TIP3P water in a cubic periodic box with 1.5 nm padding. Counterions were added to neu-tralize the system and ensure a 150 nM ionic concentration. Long-range electrostatics were treated using the Particle Mesh Ewald method with a nonbonded cutoff of 1.0 nm. Bonds involving hydrogen atoms were constrained, and hydrogen mass repartitioning (3.0 a.m.u.) was applied to enable a timestep of 5 fs. Temperature control was achieved using a Langevin integrator with a friction coefficient of 10 ps-1.

The solvated system was energy-minimized and equilibrated for 10 ns in explicit solvent at 300 K prior to production runs. Enhanced sampling simulations were performed using Simulated Tempering (ST) as implemented in the SST2 library (5). A ladder of 15 temperature rungs was defined between 300 K and 500 K. Temperature changes were attempted every 2 ps. A single continuous simulated tempering trajectory was generated for a total production time of 10 µs, during which the system dynamically sampled the predefined temperature states according to the ST algorithm.

Simulated tempering refinement of the PEP-EDIT structure using the OpenFF force field led to substantial exploration of the conformational landscape over a 10 μs production trajectory. The minimal backbone RMSD relative to the experimental bound structure (PDB ID 7F55 (6)) reached 1.2 Å, indicating that near-native conformations are accessible from the generated starting model.

Clustering of the 300 K ensemble using HDBSCAN identified 10 distinct conformational clusters. The most populated cluster (~10.6%) displayed an average backbone RMSD of 2.05 Å, while cluster 4 (~4.6%) showed an average backbone RMSD of 1.93 Å, making it the closest ensemble to the bound reference structure. Notably, all major clusters were already sampled within the first ~2 μs, indicating rapid convergence of the conformational space.

As the simulations were performed on the unbound peptide in solution, very low RMSD values were not expected when compared to the receptor-bound conformation. Nevertheless, the presence of near-native conformations among the dominant clusters demonstrates that SST2 combined with OpenFF refinement efficiently samples relevant structural states from the AI-generated starting model.

PEP-EDIT generated conformations as input for QM calculations

It is possible to use the XYZ file generated by PEP-EDIT for further modeling with quantum chemistry software packages like ORCA (7). Starting from the conformation generated by PEP-EDIT for the orphan cyclic peptide drug Cilengitide, we used the global optimizer algorithm (GOAT) (8) within ORCA to generate an ensemble of conformations. The backbone RMSD between the peptide generated with PEP-EDIT and the experimental reference (PDB 1L5G) is 1.41Å while ORCA enabled to sample more conformations with the best one achieving 0.82Å RMSD. We provide simple scripts to easily generate input files for ORCA and convert ORCA-generated XYZ conformations back to PDB for downstream structural analysis at https://github.com/alexisdougha/goat-pep.

References:

1. Murail,S., Sawmynaden,J., Zemirli,A., Jusot,M., Pietrucci,F., Chomilier,J., Tufféry,P. and Stratmann,D. (2025) Robust Conformational Space Exploration of Cyclic Peptides by Combining Different MD Protocols and Force Fields. *J. Chem. Theory Comput.*, **21**, 10018–10034.

https://doi.org/10.1021/acs.jctc.5c01123

http://www.ncbi.nlm.nih.gov/pmc/articles/PMC12661557

2. Dhillon,S. and Keam,S.J. (2019) Bremelanotide: First Approval. *Drugs*, **79**, 1599–1606.

https://doi.org/10.1007/s40265-019-01187-w

http://www.ncbi.nlm.nih.gov/pubmed/31429064

3. Eastman,P., Galvelis,R., Peláez,R.P., Abreu,C.R.A., Farr,S.E., Gallicchio,E., Gorenko,A., Henry,M.M., Hu,F., Huang,J., *et al.* (2024) OpenMM 8: Molecular Dynamics Simulation with Machine Learning Potentials. *J. Phys. Chem. B*, **128**, 109–116.

https://doi.org/10.1021/acs.jpcb.3c06662

4. Wagner,J., Thompson,M., Mobley,D.L., Chodera,J., Bannan,C., Rizzi,A., trevorgokey, Dotson,D.L., Mitchell,J.A., jaimergp, *et al.* (2025) openforcefield/openff-toolkit: 0.18.0 Runtime improvements for large molecules. 10.5281/zenodo.17525527.

https://doi.org/10.5281/zenodo.17525527

5. Stratmann,D., Moroy,G., Tuffery,P. and Murail,S. (2025) Simulated Solute Tempering 2: An Efficient and Practical Approach to Protein Conformational Sampling and Binding Events. *J. Chem. Theory Comput.*, **21**, 10705–10718.

https://doi.org/10.1021/acs.jctc.5c00950

http://www.ncbi.nlm.nih.gov/pmc/articles/PMC12661556

6. Zhang,H., Chen,L.-N., Yang,D., Mao,C., Shen,Q., Feng,W., Shen,D.-D., Dai,A., Xie,S., Zhou,Y., *et al.* (2021) Structural insights into ligand recognition and activation of

the melanocortin-4 receptor. *Cell Res.*, **31**, 1163–1175.

https://doi.org/10.1038/s41422-021-00552-3

http://www.ncbi.nlm.nih.gov/pmc/articles/PMC8563965

7. Neese,F. (2025) Software Update: The ORCA Program System—Version 6.0. *WIREs Comput. Mol. Sci.*, **15**, e70019.

https://doi.org/10.1002/wcms.70019

8. de Souza,B. (2025) GOAT: A Global Optimization Algorithm for Molecules and Atomic Clusters. *Angew. Chem. Int. Ed.*, **64**, e202500393.

https://doi.org/10.1002/anie.202500393

http://www.ncbi.nlm.nih.gov/pubmed/39959942
